# Quercetin and Quercetin-Rich Red Onion Extract Alter* Pgc-1α* Promoter Methylation and Splice Variant Expression

**DOI:** 10.1155/2017/3235693

**Published:** 2017-01-16

**Authors:** Prasad P. Devarshi, Aarin D. Jones, Erin M. Taylor, Barbara Stefanska, Tara M. Henagan

**Affiliations:** Department of Nutrition Science, Purdue University, West Lafayette, IN, USA

## Abstract

*Pgc-1α* and its various isoforms may play a role in determining skeletal muscle mitochondrial adaptations in response to diet. 8 wks of dietary supplementation with the flavonoid quercetin (Q) or red onion extract (ROE) in a high fat diet (HFD) ameliorates HFD-induced obesity and insulin resistance in C57BL/J mice while upregulating* Pgc-1α* and increasing skeletal muscle mitochondrial number and function. Here, mice were fed a low fat (LF), high fat (HF), high fat plus quercetin (HF + Q), or high fat plus red onion extract (HF + RO) diet for 9 wks and skeletal muscle* Pgc-1α* isoform expression and DNA methylation were determined. Quantification of various* Pgc-1α* isoforms, including isoforms* Pgc-1α-a*,* Pgc-1α-b*,* Pgc-1α-c*,* Pgc-1α4*, total* NT-Pgc-1α*, and* FL-Pgc-1α*, showed that only total* NT-Pgc-1α* expression was increased in LF, HF + Q, and HF + RO compared to HF. Furthermore, Q supplementation decreased* Pgc-1α-a* expression compared to LF and HF, and ROE decreased* Pgc-1α-a* expression compared to LF.* FL-Pgc-1α* was decreased in HF + Q and HF + RO compared to LF and HF. HF exhibited hypermethylation at the −260 nucleotide (nt) in the* Pgc-1α* promoter. Q and ROE prevented HFD-induced hypermethylation. −260 nt methylation levels were associated with* NT-Pgc-1α* expression only.* Pgc-1α* isoform expression may be epigenetically regulated by Q and ROE through DNA methylation.

## 1. Introduction

Peroxisome proliferator activated-receptor gamma coactivator 1 alpha* (Pgc-1α)* is a transcriptional coactivator that coordinates gene expression from the nuclear and mitochondrial genomes in order to determine mitochondrial biogenesis and function [1–4]. Environmentally induced upregulation of* Pgc-1α* plays a major role in determining skeletal muscle mitochondrial adaptations that are important in attenuating obesity and insulin resistance [1, 4–11]. Energy and nutritional status, such as the obese and diabetic state, and environmental cues, such as cold exposure, exercise training, and various dietary components, determine* Pgc-1α* expression [1, 2, 4–6, 12]. For example, high fat diet (HFD) feeding and palmitate treatment decrease* Pgc-1α* expression [6, 12, 13], whereas antiobesogenic and diabetic dietary supplements increase* Pgc-1α* expression [7, 13]. Traditionally, the molecular mechanisms regulating* Pgc-1α* have focused on posttranslational regulation of the protein affecting protein stability and its ability to bind target genes to induce transcriptional activation [1, 14]; however, more recent investigations have shown a major role of epigenetic modifications to the* Pgc-1α* promoter to play a role in its transcriptional regulation and mRNA expression in response to environmental and energy/nutritional inputs [5, 6, 13, 15, 16]. Exercise training, dietary fat content, long chain fatty acid exposure, and the presence of diabetes and overweight or obesity have all been shown to epigenetically regulate* Pgc-1α* mRNA expression and downstream mitochondrial adaptations in skeletal muscle [5, 6, 13, 15, 16].

Not only is* Pgc-1α* regulated posttranslationally and transcriptionally, but also several* Pgc-1α* isoforms with novel and specific activities have been identified [17–19]. The various expression patterns of these* Pgc-1α* isoforms are also regulated by environmental stimuli, including cold exposure and exercise training, and by the obese state [15, 17–22]. To date, more than ten isoforms of* Pgc-1α* are known to exist, arising from a combination of distinct promoter start sites and alternative splicing [23]. The most extensively studied isoforms in skeletal muscle are* Pgc-1α-b, Pgc-1α-c, Pgc-1α4, *the* n*-truncated splice variant* NT-Pgc-1α*, and the full length variant* FL-Pgc-1α* [17–19, 21, 23, 24]. While many isoforms possess overlapping functions, several have been shown to have distinct functions in skeletal muscle [23]. For example, exercise training preferentially upregulates expression of the isoforms arising from the alternative, distal promoter region, including isoforms* Pgc-1α-b, Pgc-1α-c, Pgc-1α4*, total* NT-Pgc-1α, *and* FL-Pgc-1α* [23]. Additionally,* Pgc-1α-a*,* Pgc-1α-b, Pgc-1α-c, *total* NT-Pgc-1α, *and* FL-Pgc-1α* are all known to play a role in exercise-induced mitochondrial adaptations in skeletal muscle [19]. Distinct from the other exercise-induced isoforms,* Pgc-1α4 *plays a role in promoting muscle hypertrophy [21]. More recently, various isoforms of* NT-Pgc-1α (NT-Pgc-1α-a, NT-Pgc-1α-b, *and* NT-Pgc-1α-c)*, derived from either the proximal or distal promoters, have been characterized. All* NT-Pgc-1α* isoforms have been shown to be induced during either high intensity* (NT-Pgc-1α-a)* or low intensity* (NT-Pgc-1α-b *and* NT-Pgc-1α-c) *exercise, contributing to upregulation of total* NT-Pgc-1α* that occurs at all intensities of exercise [19]. Additionally, all* NT-Pgc-1α* isoforms and total* NT-Pgc-1α* are upregulated in skeletal muscle in response to AICAR treatment, a pharmacological activator of AMPK known to increase mitochondrial biogenesis and fatty acid oxidation in skeletal muscle [19]. Collectively, the isoforms most notably known to play roles in skeletal muscle mitochondrial adaptations to date include* Pgc-1α-a, Pgc-1α-b, Pgc-1α-c, Pgc-1α4, *total* NT-Pgc-1α*, and* FL-Pgc-1α*. Expression patterns of these various splice variants may have the ability to prevent disease, as overexpression of total* NT-Pgc-1α* is also known to attenuate HFD-induced obesity [25]. Interestingly, recent studies show that epigenetics may play a role in determining mRNA splicing [26] in addition to its role in determining transcription initiation and −1 nucleosome positioning may play a role in determining* FL-Pgc-1α* and total* NT-Pgc-1α* expression in skeletal muscle in relation to cardiovascular disease during overweight and obesity [15]. Thus,* Pgc-1α* isoform expression may be epigenetically regulated during disease and in response to environmental stimuli, including dietary inputs.

Quercetin (Q) is a bioflavanoid that protects against mitochondrial dysfunction and attenuates HFD-induced obesity and insulin resistance when supplemented in a HFD at low concentrations [7, 27–32]. We have recently shown that a low dose (50 ug/day) of dietary Q supplementation or dietary supplementation with red onion extract (ROE), containing equivalent amounts of Q glycosides (50 ug/day), increases skeletal muscle mitochondrial number and function, leading to more complete fatty acid oxidation [7, 32], similar to the effects of exercise training on skeletal muscle. However, the effects of Q or ROE appear to occur through regulation of differential molecular mechanisms at the level of mitochondrial gene transcription [32], a process that may be controlled by the transcriptional coactivation abilities of* Pgc-1α*.

To investigate the role of dietary fat, purified dietary Q, and ROE in determining* Pgc-1α* DNA methylation in skeletal muscle in association with HFD-induced obesity and insulin resistance and elaborate on how these epigenetic effects associate with splice variant expression, we used diet-induced obese C57BL/6J mice as a model system. We hypothesized that the mitochondrial gene expression patterns previously observed and published [32] would be associated with differential diet-induced methylation patterns in the PGC-1 promoter and regulation of those specific* Pgc-1α* isoforms known to play a role in skeletal muscle mitochondrial adaptations in response to environmental stimuli [17, 18, 21, 25].

## 2. Materials and Methods

### 2.1. Animals and Diets

The protocols for animal and diets have been previously published [32]. Briefly, ROE was prepared as previously described [32] and formulated into a purified high fat diet (Research Diets D12451, 45% kcal fat) to yield 17 mg/kg of quercetin equivalents [32]. 5-week-old C57BL/6J mice (Jackson Laboratories, Bar Harbor, MN, USA) were weaned onto low fat diet (LF; Research Diets 12450B, 10% kcal fat) for 1 week and then randomized into 4 dietary treatment groups (*N* = 10/group): LF (Research Diets 12450B, 10% kcal fat); high fat (HF; Research Diets D12451, 45% kcal fat); HF + Q (Research Diets D08072305, 45% kcal fat) with 17 mg/kg quercetin aglycone (Enzo Life Technologies ALX-385-001-G005; Farmingdale, NY, USA); or HF + RO (Research Diets D08072306). Mice were fed respective diets for 9 wks, during which time 48 h food consumption and body weight and composition via nuclear magnetic resonance (Bruker Minispec, Billerica, MA, USA) were assessed weekly. Mice were euthanized after 9 wks of feeding and quadriceps muscle was extracted and snap frozen in liquid nitrogen for further analyses. All experiments were reviewed and approved by the Pennington Biomedical Research Center Institutional Animal Care and Use Committee.

### 2.2. DNA Isolation and Bisulfite Treatment

To provide a more homogenous sample, frozen quadriceps muscle was ground under liquid nitrogen using a mortar and pestle. Ground muscle was then used for the extraction of genomic DNA with a DNeasy Blood and Tissue Kit (Qiagen 69581) via the manufacturer's protocol. The quantity and quality of the gDNA extracted were determined by spectrophotometry using a NanoDrop (Thermo Scientific, Wilmington, DE, USA). Approximately 200 ng of gDNA was subjected to bisulfite conversion using the EpiTect Bisulfite Kit (Qiagen 59104) via the manufacturer's protocol.

### 2.3. Bisulfite Sequencing

Bisulfite sequencing was performed on the Qiagen PyroMark Q24 platform (Qiagen 9001514). Bisulfite converted gDNA was PCR amplified using the EpiTect MSP kit (Qiagen) and biotinylated primers targeting the* Pgc-1α* gene. Human primer sequences that amplify the region of the* Pgc-1α* promoter surrounding the −260 nucleotide site have been previously published [6]. Here, primer sequences (IDT) that amplified and biotinylated the corresponding region within the mm10 reference genome that surrounds the methylation site were used with the PyroMark PCR kit (Qiagen 978703) per the manufacturer's instructions. Biotinylated DNA then subjected to pyrosequencing on the PyroMark Q24 (Qiagen 9001514) with PyroMark Gold Reagents (Qiagen), following the manufacturer's instructions. PyroMark analysis software was used to determine the DNA methylation percentage at the −260 nucleotide (nt).

### 2.4. RNA Isolation

Total RNA was extracted from quadriceps muscle tissue using Tri-Reagent (Molecular Research Center, Cincinnati, OH, USA) followed by further purification with a RNeasy mini kit (Qiagen, Valencia, CA, USA), as previously described [32]. The quantity and quality of the RNA were analyzed by spectrophotometry (NanoDrop, ND-1000, Thermo Scientific, Wilmington, DE, USA). RNA was reverse transcribed into a cDNA library using M-MLV reverse transcriptase (Promega, Madison, WI, USA).

### 2.5. qRT-PCR

qRT-PCR was performed using previously published primer pairs that targeted various* Pgc-1α* isoforms [17, 18, 21]. Primer sequences are shown in Table 1. All samples were run in duplicate on the ABI QuantStudio 6 Flex platform (Applied Biosystems, Foster City, CA, USA) using SyBR Green MasterMix (Applied Biosystems, Foster City, CA, USA). Gene expression was analyzed using a standard curve and normalization to cyclophilin B as the endogenous control. Data are expressed as arbitrary units (AU).

### 2.6. Statistical Analysis

The data were analyzed with GraphPad Prism 5.0 statistical analysis software. Results are expressed as means ± standard error. Food consumption and body weight composition parameters were analyzed by repeated measures ANOVA. All other measurements were analyzed by one-way ANOVA. A Tukey test was used post hoc as necessary. A* P* value < 0.05 was used to determine significance.

## 3. Results

### 3.1. Mouse Dietary Model

**Figure 1:**
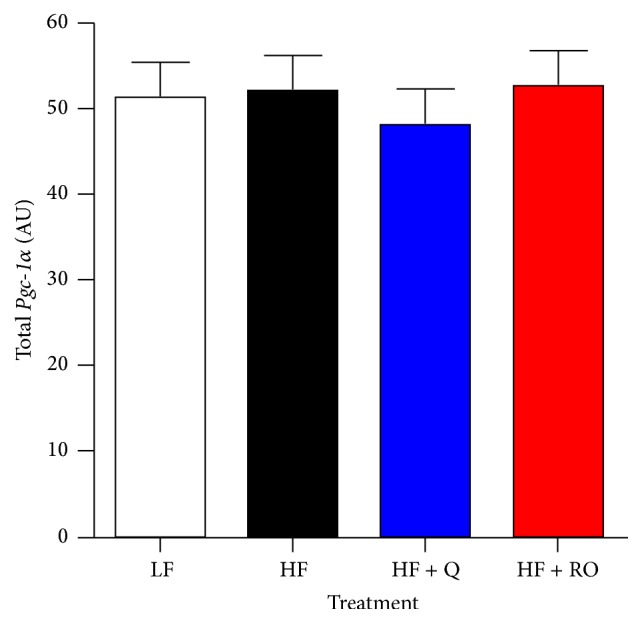
*Pgc-1α* mRNA was measured by qRT-PCR in skeletal muscle samples of mice fed a low fat (LF), high fat (HF), high fat plus quercetin (HF + Q), or high fat plus red onion extract (HF + RO) diet for 9 wks. Values are shown as means ± SEM in arbitrary units (AU).

We have previously published the phenotypic data showing that 50 ug/g of dietary quercetin or ROE supplementation attenuates HFD-induced obesity and insulin resistance in C57BL/6J mice [32]. Interestingly, the antiobesogenic and antidiabetic effects of quercetin and ROE occur in association with ~50% increase in skeletal muscle mitochondrial number and more complete beta oxidation in skeletal muscle [32]. In our previous studies, C57BL/6J mice received Q supplementation for 8 wks and this resulted in an increase in skeletal muscle* Pgc-1α* expression in skeletal muscle [32]. Thus, in the present study, we also aimed to determine if ROE acts similarly to Q to upregulate* Pgc-1α* in association with previously observed beneficial mitochondrial adaptations. Contrary to our previous findings, in the present study we found that, after 9 wks of supplementation, neither Q nor ROE altered total* Pgc-1α* expression despite the previously published improvements in skeletal muscle mitochondrial number in these animals (Figure 1).

### 3.2. *Pgc-1α* Isoform Expression

**Figure 2:**
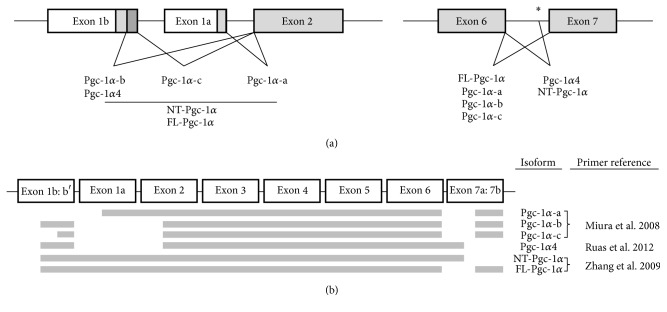
(a) Schematic structures of the 5′ and 3′ regions of* Pgc-1α* are shown for each isoform. The 5′ schematic was slightly adapted from Miura et al. 2008. The proximal (exon 1a) or alternate (exon 1b) promoters can be spliced to exon 2. Within exon 1b, upstream (exon 1b) or downstream (exon 1b′) splicing to exon 2 can occur. Splicing also occurs between exons 6 and 7 near the 3′ end of the gene. Exon 6 can be spliced directly to exon 7, at two separate regions, 7a or 7b. Alternatively, exon 6 can be spliced to the intronic region preceding exon 7 to result in a truncated form of* Pgc-1α*. Total* NT-Pgc-1α*,* FL-Pgc-1α*, and total* Pgc-1α* all contain exons 1b:1b′, 1a, and 2 as indicated. Total* Pgc-1α* contains both exons 6 and 7, as indicated. (b) Schematic representation of the start and stop of each* Pgc-1α* isoform is shown. Primer pairs have previously been designed and published, and the corresponding reference for each pair is indicated. *∗* is the alternative splice site between exons 6 and 7.

**Figure 3:**
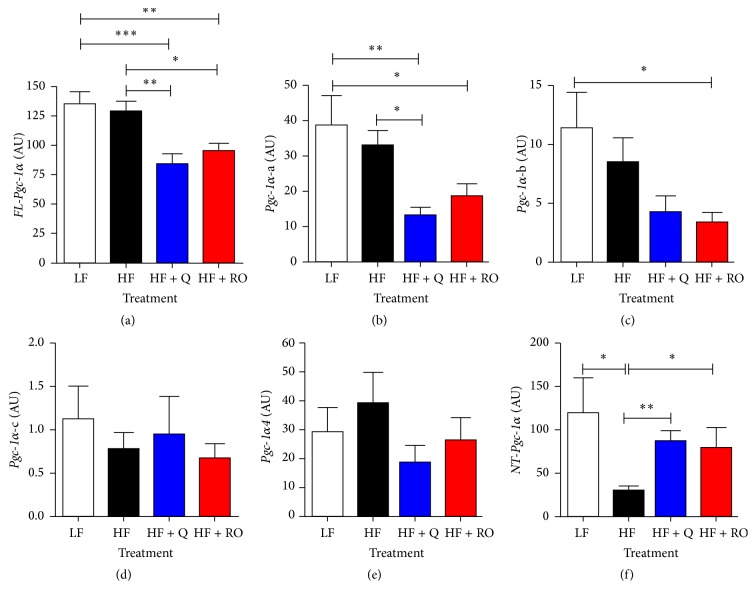
Expressions of (a)* FL-Pgc-1α*, (b)* Pgc-1α-a*, (c)* Pgc-1α-b*, (d)* Pgc-1α-c*, (e)* Pgc-1α4*, and total* NT-Pgc-1α* isoforms were measured by qRT-PCR in skeletal muscle samples of mice fed a low fat (LF), high fat (HF), high fat plus quercetin (HF + Q), or high fat plus red onion extract (HF + RO) diet for 9 wks. Values are shown as means ± SEM in arbitrary units (AU). *∗* denotes significant difference with* P* < 0.05, ^*∗∗*^*P* < 0.01, and ^*∗∗∗*^*P* < 0.001.

Several splice variants of* Pgc-1α* have recently been described, with some splice variants showing overlapping transcriptional coactivator activities and downstream physiological changes in metabolic and mitochondrial adaptations in response to environmental inputs [17–19, 21]. Due to the lack of change observed in total* Pgc-1α* (Figure 1) despite the significant increase in mitochondrial number, we aimed to determine if Q or ROE may act to upregulate specific splice variants of* Pgc-1α* using our model with known skeletal muscle mitochondrial adaptations [7, 32]. A schematic of the various* Pgc-1α* variants measured in the present study is shown in Figure 2.* Pgc-1α-a*,* Pgc-1α-b, Pgc-1α-c*, and* Pgc-1α4* differ in their N-termini due to transcription starting within either the distal, alternative (exon 1b), or proximal (exon 1a) promoters and also due to alternative splicing of exon 1b to produce exon 1b′, whereas* NT-Pgc-1α* and* FL-Pgc-1α* both contain the full exonic regions from 1b, 1b/, 1a, and 2 (Figure 2(a)). In addition to splicing at the 5′ region,* Pgc-1α* may undergo further splicing at the 3′ end, resulting in differential C-termini. As depicted in Figure 2(a),* Pgc-1α-a*,* Pgc-1α-b, Pgc-1α-c, *and* FL-Pgc-1α* contain exons 6 and 7 within the respective, mature transcripts, whereas* Pgc-1α4 *and* NT-Pgc-1α* undergo alternative splicing resulting in an insertion between exons 6 and 7 and subsequent termination of translation before exon 7. Interestingly, both HF + Q and HF + RO exhibited decreases in* FL-Pgc-1α* compared to LF and HF groups (Figure 3(a)). HF + Q and HF + RO also showed a decrease in* Pgc-1α-a *compared to LF and HF, although the decrease in HF + RO compared to HF did not reach statistical significance (Figure 3(b)). HF + RO but not HF + Q showed decreased* Pgc-1α-b *expression compared to LF (Figure 3(c)). No change was observed in any groups for* Pgc-1α-c *or* Pgc-1α4* (Figures 3(d) and 3(e)). Interestingly, the only differences observed between LF and HF occurred with a decrease in total* NT-Pgc-1α* expression (Figure 3(f)). HFD-induced decreases in total* NT-Pgc-1α* were prevented in HF + Q and HF + RO, who showed a significant increase in total* NT-Pgc-1α* expression compared to HF (Figure 3(f)).

### 3.3. *Pgc-1α* DNA Methylation at the −260 Nucleotide

**Figure 4:**
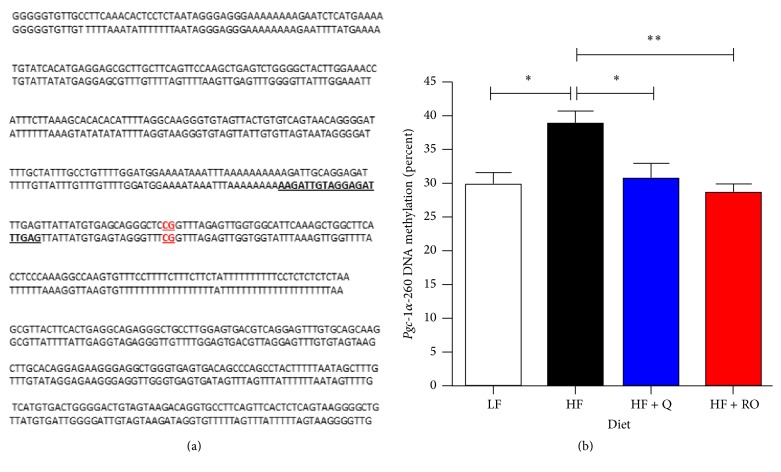
Bisulfite sequencing of the* Pgc-1α* promoter was used to determine DNA methylation levels at the −260 nt. The original (top line) and bisulfite converted (corresponding bottom line) sequences are shown in (a). The bold and underlined region within the bisulfite converted sequence indicates where the pyrosequencing primer is located. The red highlighted, bold, and underlined region is the regulatory −260 nt that undergoes DNA methylation. The percent of DNA methylation present at the −260 nt in skeletal muscle is shown as the mean ± SEM for low fat (LF), high fat (HF), high fat plus quercetin (HF + Q), and high fat plus red onion extract (HF + RO) animals after 9 wks of feeding the respective diets. *∗* denotes significant difference with* P* < 0.05 and ^*∗∗*^*P* < 0.01.

*Pgc-1α* has recently been shown to be hypermethylated in the skeletal muscle of type 2 diabetic individuals, in response to palmitate and oleate treatment of myocytes in vitro and by 10 wks of HFD feeding in C57BL/6J mice [6, 13]. Specific DNA methylation at the  −260 nt is sufficient to decrease* Pgc-1α* expression and leads to detrimental skeletal muscle mitochondrial adaptations [6, 13]. To determine if Q and RO may work to ameliorate HFD-induced skeletal muscle mitochondrial maladaptations through epigenetic regulation of* Pgc-1α*, bisulfite sequencing of the* Pgc-1α* promoter was performed, specifically measuring the percentage of DNA methylation at the regulatory −260 nt site. The bisulfite converted DNA sequence and location of the pyrosequencing primer and known DNA methylation site are shown in Figure 4(a).* Pgc-1α* −260 nt methylation was significantly increased in HF compared to LF (Figure 4(b)). Q and ROE supplementation at 50 ug/day prevented the HFD-induced increases in DNA methylation, leading to a percentage of methylation in these groups that was similar to that observed in LF animals (Figure 4(b)).

## 4. Discussion

DNA methylation of the* Pgc-1α* promoter decreases skeletal muscle* Pgc-1α* expression and mitochondrial number and function, both of which may be important in determining insulin sensitivity during obesity [5, 6, 8, 9, 13, 33, 34]. Environmental inputs, such as diet and exercise, alter* Pgc-1α* expression to determine skeletal muscle mitochondrial adaptations [1, 2, 4, 14]. Dietary supplementation with the flavonoid Q and/or the botanical extract from red onions prevents HFD-induced obesity and insulin resistance by increasing skeletal muscle* Pgc-1α* expression and mitochondrial function and number [7, 31, 32]. Due to their effects on* Pgc-1α* and mitochondrial adaptations and the ability of* Pgc-1α* to be epigenetically regulated, it is possible that the effects of Q and ROE occur through an epigenetic mechanism involving DNA methylation. Here, we show that 9 wks of HFD feeding, which causes increases in adiposity and insulin resistance in C57BL/6J mice [32], leads to hypermethylation at the −260 nt of the* Pgc-1α* promoter. Both Q and ROE attenuate HFD-induced obesity and insulin resistance while preventing HFD-induced hypermethylation of the −260 nt in* Pgc-1α*. Although we have previously found that 50 ug/day of Q supplementation upregulates skeletal muscle* Pgc-1α* in association with increased mitochondrial function in the form of more complete beta oxidation of fatty acids [7] and the mice in the present study showed an ~50% increase in skeletal muscle mitochondrial number [32], in the present study we observed no difference in* Pgc-1α* expression (measured as the total* Pgc-1α* expression) between HF and HF + Q or HF + RO.

Importantly, the effects of dietary Q supplementation on skeletal muscle* Pgc-1α* expression and mitochondrial adaptations occur in a dose- and time-dependent manner [7, 31]. Our previous study showed an increase in* Pgc-1α* at 8 wks of supplementation [7] and, in the present study,* Pgc-1α* was measured after 9 wks of supplementation. Despite the lack of increase in total* Pgc-1α*, we continued to observe Q- and ROE-induced increases in energy expenditure and mitochondrial number [32], which may partially be the result of prior* Pgc-1α* upregulation at 8 wks. However, mitochondrial turnover occurs rapidly, ranging from ~1 to 17 days in a tissue-specific manner, and this turnover rate can be increased by dietary interventions, such as caloric restriction [35, 36]. Thus, one would expect that if the effects of Q and ROE are no longer beneficial after 9 wks due to the lack of change in total* Pgc-1α* expression, the continuous impetus of the HFD would lead to similar skeletal muscle physiologies in HF, HF + Q, and HF + RO groups; yet at 9 wks, we still observed increased mitochondrial number [32] and decreased* Pgc-1α* methylation in HF + Q and HF + RO. Thus, it appears that although total* Pgc-1α* remains unchanged, other mechanisms may compensate for this loss of* Pgc-1α* upregulation to perpetuate beneficial mitochondrial adaptations in HF + Q and HF + RO.

Interestingly, DNA methylation has been shown to drive mRNA splicing and* Pgc-1α* has several known isoforms that result from alternative splicing and alternate promoter start sites [17–19, 21, 26]. These isoforms have unique and complementary or overlapping functions, specific to each variant [17, 18, 21, 25]. Thus, it is possible that the effects of Q and ROE may be due to epigenetic regulation of mRNA splicing and subsequent alterations in* Pgc-1α* splice variant expression. Indeed, we show here that Q and ROE decreased expression of* FL-Pgc-1α* and isoform a, without changing isoforms b, c, or 4. Additionally, Q but not ROE decreased* Pgc-1α* isoform B in comparison to LF but HF. Here, none of the treatments had an effect on* Pgc-1α* isoforms 4 and c. The only isoform shown to decrease after 9 wks of HFD feeding was* NT-Pgc-1α*; and, both Q and ROE were able to prevent this HFD-induced decrease. These results are consistent with changes in DNA methylation status at the −260 nt, with HF increasing in methylation but HF + Q and HF + RO showing similar levels of methylation as LF. Thus at 9 wks of feeding, it is possible that the beneficial effects of Q and ROE on mitochondrial number and function are mediated via upregulation of* NT-Pgc-1α* and that isoform expression is dependent on epigenetic regulation of* Pgc-1α*. Indeed,* NT-Pgc-1α* has known complementary and overlapping functions to* FL-Pgc-1α* and may compensate for loss of* FL-Pgc-1α* or other isoforms [17, 25]; and it has recently been shown that* Pgc-1α* isoform expression is epigenetically regulated by histone methylation in response to exercise, with expression of* Pgc-1α-b* and of* Pgc-1α-c* but not of* Pgc-1α-a* being upregulated in conjunction with increased histone methylation in the promoter of exon 1b compared to exon 1a [16].

Here, we measured the percentage of −260 nt DNA methylation, a methylation site known to regulate* Pgc-1α* expression and skeletal muscle mitochondrial adaptations which is located within the proximal promoter upstream of exon 1a. Given that Q and ROE decreased* Pgc-1α* isoforms* Pgc-1α-a* and* Pgc-1α-b* without changing* Pgc-1α-c*, and* Pgc-1α-a* includes exon 1a,* Pgc-1α-b* includes exon 1b, and* Pgc-1α-c* includes exon 1b′, it appears that −260 nt methylation does not play a role in regulating 5′ splicing or transcription initiation within* Pgc-1α* proximal versus alternative, distal promoters. This is consistent with the recent publication by Lochmann et al. 2015, who similarly showed that* Pgc-1α* methylation was not related to* Pgc-1α* isoform a, b, or c expression [16]. Interestingly, total* Pgc-1α* expression was also not associated with DNA methylation at the −260 nt. However, it is unclear in previous publications what isoforms of* Pgc-1α* were measured and correlated with −260 nt DNA methylation. It is possible that previous reports use primers that measure isoforms which are differentially spliced at the 3′ prime end; as in the present study, although we did not observe associations between −260 nt methylation and 5′ splicing or specific promoter initiation, we did see alterations in isoform expression based on splicing at the 3′ region in association with DNA methylation levels at the −260 nt. Notably,* FL-Pgc-1α* decreased in HF + Q and HF + RO and total* NT-Pgc-1α* increased. Although alternative splicing that produces these isoforms occurs at exons 6 and 7 and the intronic region between these exons [17] and DNA methylation occurs within the promoter region, promoter structure is known to be important for alternative splicing [26]. Promoter methylation regulates splicing through a complex process involving epigenetic modifications and promoter occupation of transcriptional activators [26]. DNA methylation not only alters transcription factor binding, but also directly regulates alternative splicing by recruiting RNA-binding proteins that can be transferred to the mRNA to alter the splicing pattern [26]. Although the present study is limited by measuring only the −260 nt methylation status, others have shown that methylation at the −260 nt occurs when the entire* Pgc-1α* promoter is hypermethylated [6]. Thus, hyper- and hypomethylation of the −260 nt may serve as a surrogate measure to indicate methylation status across the entire promoter or gene, whose methylation may play a role in determining alternative splicing and isoform expression.

In conclusion, Q and ROE show similar effects in preventing* Pgc-1α* −260 nt methylation induced by HFD feeding, upregulating total* NT-Pgc-1α* splice variant expression in skeletal muscle. Thus, the perpetuating beneficial effects of Q and ROE on body weight, body composition, energy expenditure, insulin sensitivity, and skeletal muscle mitochondrial number and function in mice after 9 wks of supplementation [32] may be mediated via upregulation of total* NT-Pgc-1α*. There is a further need to study the functions and epigenetic regulation of total* NT-Pgc-1α* and other isoforms of* Pgc-1α* to determine their specific roles in regulating skeletal muscle adaptations and contribute to a lean, insulin sensitive state.

## 5. Conclusions

HFD causes* Pgc-1α* hypermethylation at the −260 nt in the* Pgc-1α* proximal promoter in conjunction with alterations in* Pgc-1α* splice variant expression in skeletal muscle. Q and ROE similarly prevent HFD-induced hypermethylation of* Pgc-1α* and increase* NT-Pgc-1α* expression in skeletal muscle. Q- and ROE-induced upregulation of* NT-Pgc-1α* may occur through an epigenetic mechanism and may be sufficient to increase skeletal muscle mitochondrial number and complete beta oxidation of fatty acids, contributing to attenuation of obesity and insulin resistance.

## Figures and Tables

**Table 1:** * Pgc-1α* isoform primer pair sequences are shown for the forward (F) and reverse (R) primers used to measure isoform expression via qRT-PCR. Primer pairs have previously been reported by Miura et al. 2008, Ruas et al. 2012, and Zhang et al. 2009.

Primer target	Primer sequence (5′ to 3′)
*Pgc-1α-a *F	GCTTGACTGGCGTCATTCG
*Pgc-1α-a *R	ACAGAGTCTTGGCTGCACATGT
*Pgc-1α-b *F	GACATGGATGTTGGGATTGTCA
*Pgc-1α-b *R	ACCAACCAGAGCAGCACATTT
*Pgc-1α-c* F	AGTGACATGGATGTTGGGATTG
*Pgc-1α-c* R	GAATGCCTCCGGTTACTCACTT
*Pgc-1α4* F	TCACACCAAACCCACAGAAA
*Pgc-1α4* R	CTG GAA GAT ATG GCA CAT
*FL-Pgc-1α*F	TGCCATTGTTAAGACCGA
*FL-Pgc-1α*R	CCAGAGTCACCAAATGACC
total *NT-Pgc-1α* F	TGCCATTGTTAAGACCGA
total *NT-Pgc-1α* R	CCATATCTTCCAGTGACC
total *Pgc-1α* F	TGATGTGAATGACTTGGATACAGACA
total *Pgc-1α* R	GCT CAT TGT TGT ACT GGT TGG ATA TG
